# Sex differences in neurogenic hypertension: estrogen modulation of NADPH oxidase signaling in the rostral ventrolateral medulla

**DOI:** 10.1186/s13293-026-00966-x

**Published:** 2026-08-14

**Authors:** Jing Jin, Umm-E. Kalsoom, Yao Su, Wanying Meng, Li Yang, Feng Wang, Changhao Wu, Zhifeng Dong, Qin Wu

**Affiliations:** 1 Northern Jiangsu Cardiovascular Disease Prevention and Treatment Research Institute, Jiangsu University, Yancheng, 224000 China; 2 School of Medicine, Jiangsu Medical College, Yancheng, 224005 China; 3 International Genome Center, Jiangsu University, Zhenjiang, 212013 China; 4 Yancheng Third People’s Hospital, the affiliated hospital of Jiangsu Medical College, Yancheng, 224001 China; 5 School of International Education, Xuzhou Medical University, Xuzhou, 221004 China; 6 College of Marine and Bioengineering, Yancheng Institute of Technology, Yancheng, 224051 China; 7 School of Biosciences and Medicine, University of Surrey, Guildford, UK

**Keywords:** Neurogenic hypertension, Rostral ventrolateral medulla, Oxidative stress, Estrogen, Sex differences, NADPH oxidase

## Abstract

Neurogenic hypertension is characterized by sustained sympathetic activation arising from dysregulation within the central autonomic network. The rostral ventrolateral medulla (RVLM) is a major sympathetic premotor region that integrates reflex, neurohumoral, inflammatory, and metabolic signals. Oxidative stress within the RVLM can increase neuronal excitability, enhance glutamatergic transmission, weaken nitric oxide-dependent GABAergic inhibition, and promote sympathetic activation. Nicotinamide adenine dinucleotide phosphate (NADPH) oxidases are important sources of reactive oxygen species in this region. Direct anatomical evidence is the strongest for components of the NADPH oxidase 2 (NOX2) complex, whereas the cellular distribution and functional roles of Nox1, Nox4, and other isoforms remain less clearly defined. Current evidence more strongly supports RVLM Nox-related oxidative stress as a mechanism that amplifies and maintains established hypertension than as an independent initiator of chronic hypertension.

Sex and ovarian hormone status modulate these redox and autonomic mechanisms. Estrogen signaling can influence neuronal excitability, inflammation, and oxidative balance, but its effects depend on receptor subtype, endocrine state, exposure pattern, age, and hypertensive background. Estradiol replacement after ovarian hormone loss may reduce RVLM oxidative stress, whereas prolonged exposure in intact cycling animals may increase superoxide production and Nox-related gene expression. Human studies also associate menopause with altered blood pressure, arterial stiffness, sympathetic activity, and baroreflex regulation, although direct evidence for estrogen receptor and Nox signaling within the human RVLM remains lacking. This review summarizes current evidence and highlights priorities for mechanistic and translational research.

## Introduction

Hypertension remains a major global health problem and an important cause of cardiovascular morbidity and mortality, substantially increasing the risks of stroke, myocardial infarction, and kidney disease. Large-scale analyses from the Global Burden of Disease Study identify elevated blood pressure as one of the leading contributors to global mortality and disability [1]. Hypertension is mainly considered a peripheral vascular and renal regulatory disorder, but the central nervous system is also involved in its development and maintenance. Dysfunction of the central autonomic nervous pathway can lead to continuous excitation of the sympathetic nervous system, underlying neurogenic hypertension.

Cardiovascular regulation depends on an interconnected central autonomic network involving hypothalamic, brainstem, spinal, cortical, and limbic regions. These structures integrate reflex, neurohumoral, inflammatory, metabolic, and behavioral information and coordinate autonomic output to maintain blood pressure homeostasis. Increased sympathetic activity is frequently observed in hypertension and contributes to elevated peripheral vascular resistance, renal sodium and water retention, increased cardiac workload, and vascular remodeling [2]. Within this network, the rostral ventrolateral medulla (RVLM) is a major sympathetic premotor region that converts convergent central inputs into descending vasomotor drive.

Oxidative stress within central cardiovascular regions has emerged as an important mechanism of sympathetic dysregulation. Reactive oxygen species (ROS) participate in physiological intracellular signaling, but excessive or poorly controlled ROS production can alter neuronal excitability, synaptic transmission, and intracellular signaling pathways. The NADPH oxidase family of enzymes is an important, regulated source of ROS in central autonomic regions. Within the RVLM, excessive redox signaling may strengthen excitatory transmission, weaken inhibitory control, and increase the activity of presympathetic neurons [3].

The prevalence of hypertension varies significantly by age and sex. Global population studies show that the prevalence of hypertension increases with age, exhibits different trends between sexes, and some research suggests that menopause may be associated with a greater increase in systolic blood pressure in some women [4–6]. Premenopausal women generally have lower blood pressure and a lower prevalence of hypertension than age-matched men, but this difference diminishes after menopause [4]. These epidemiological patterns suggest that the loss of ovarian hormone-sensitive regulation may contribute to changes in blood pressure after menopause, although they do not establish a direct causal relationship. Potential contributing mechanisms include alterations in vascular function, autonomic regulation, renal sodium handling, metabolism, and inflammation [8]. Experimental studies of female and male differences in Ang II-sensitive oxidative and autonomic pathways provide such mechanistic context, alongside the vascular, renal, metabolic, and environmental factors that may shape the differences in the human population [7].

The central mechanisms through which biological sex and ovarian hormones influence blood pressure are still being resolved [8]. The RVLM represents a potential site at which estrogen receptor signaling, Nox-related redox regulation, neuronal excitability, and sympathetic output converge [3, 9–11]. Estrogenic actions vary across experimental conditions. Estradiol replacement after ovarian hormone loss and prolonged estradiol exposure in intact animals can produce different redox and cardiovascular outcomes [10, 12]. Receptor-selective stimulation, aging, and hypertension-associated receptor remodeling may further modify these responses [9, 11, 13].

This review explores how the organization of the central autonomic nervous system, Nox-related redox signals within the RVLM, and the estrogen receptor pathway interact to influence sympathetic regulation and differences in susceptibility to hypertension between men and women. It focuses on the anatomy and neurochemical organization of presympathetic neurons in the RVLM, the pathophysiological significance of oxidative stress in the RVLM, the mechanisms of estrogen sensitivity in upstream autonomic regions, the direct effects of estrogen within the RVLM, and the translational significance of menopause and menopausal hormone therapy (MHT). This review evaluates existing evidence from a condition-dependent perspective, considering factors such as endocrine status, ligand type, receptor subtype, dosage, time of action, age, central site of action, and disease model, rather than simply generalizing estrogen to have a uniform protective effect.

## Central autonomic network regulation of blood pressure and neurogenic hypertension

### Central autonomic network in blood pressure regulation

Arterial pressure is regulated by an interconnected central autonomic network spanning higher brain regions, the hypothalamus, brainstem, and spinal cord. This network integrates baroreceptor, chemoreceptor, neurohumoral, inflammatory, metabolic, and behavioral signals and adjusts sympathetic and parasympathetic outflow to maintain cardiovascular homeostasis [2, 3].

Within this network, cortical and limbic regions shape cardiovascular responses to stress; the paraventricular nucleus of the hypothalamus (PVN) and other hypothalamic nuclei integrate neuroendocrine and autonomic signals; circumventricular organs (CVOs) detect circulating Ang II, cytokines, and osmotic cues; and pathways involving the nucleus tractus solitarius (NTS), caudal ventrolateral medulla (CVLM), and RVLM process cardiovascular reflex information [2, 3]. The RVLM serves as a major premotor output region that converts these convergent influences into sympathetic vasomotor drive directed toward sympathetic preganglionic neurons in the spinal intermediolateral column [2, 3].

**Fig. 1:**
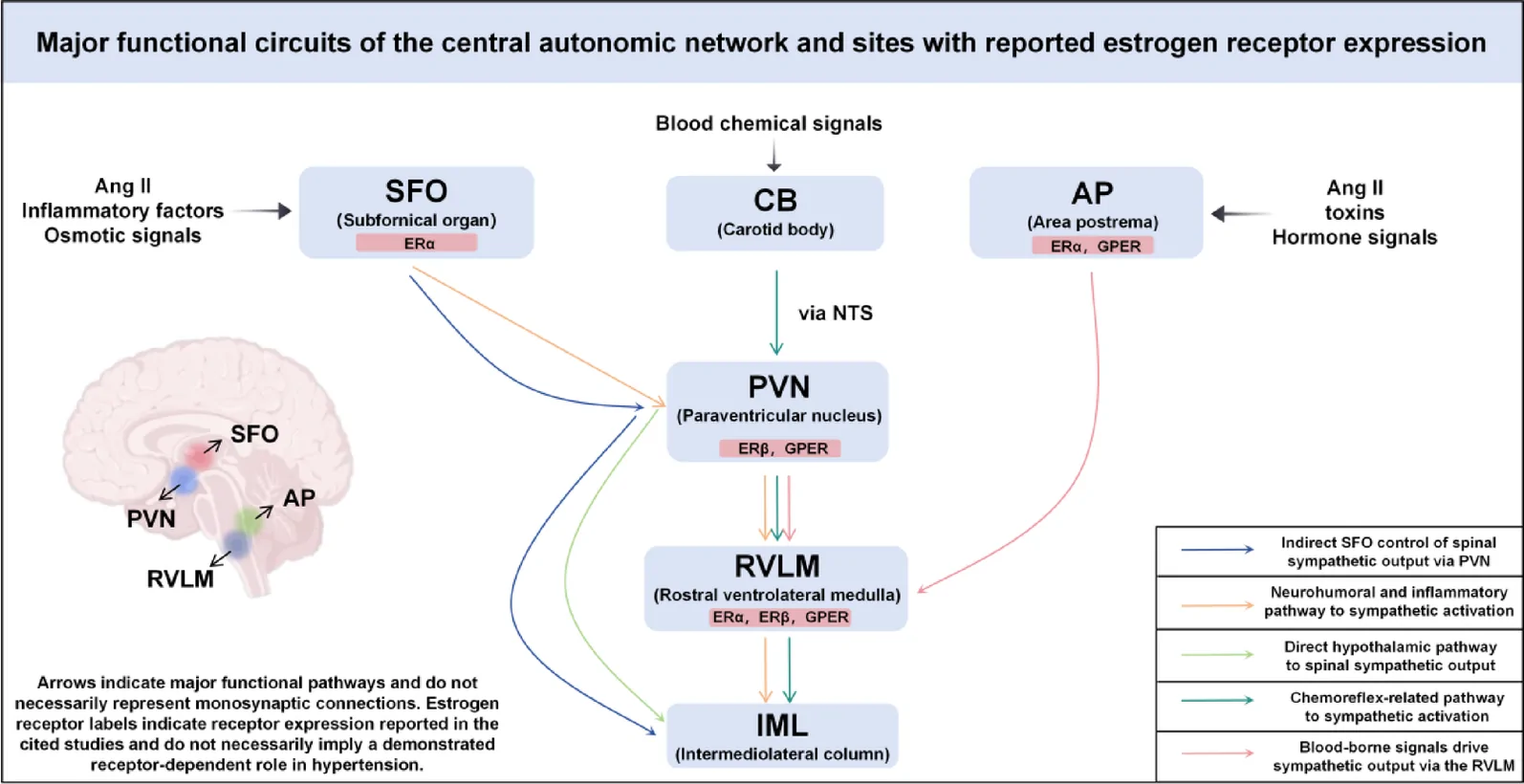
**Major functional circuits of the central autonomic network and sites with reported estrogen receptor expression.** The schematic summarizes the SFO–PVN–RVLM–IML, SFO–PVN–IML, CB afferent–NTS–PVN–RVLM–IML, and AP–RVLM–IML pathways involved in the integration of circulating neurohumoral, inflammatory, osmotic, and peripheral chemoreceptor signals and the regulation of spinal sympathetic output [2, 3, 14-27]. Estrogen receptor labels are based on reported expression of ERα in the SFO [28], ERβ in the PVN [29], GPER in the PVN [30], ERα, ERβ, and GPER in the RVLM [9, 11], and ERα and GPER in the AP [31, 32]. Arrows indicate major functional pathways and do not necessarily represent monosynaptic connections. Reported receptor expression does not necessarily indicate a demonstrated receptor-dependent role in hypertension. This original schematic was created by the authors based on the cited studies. Abbreviations: AP, area postrema; CB, carotid body; ERα, estrogen receptor alpha; ERβ, estrogen receptor beta; GPER, G protein-coupled estrogen receptor; IML, intermediolateral column of the spinal cord; NTS, nucleus tractus solitarius; PVN, paraventricular nucleus of the hypothalamus; RVLM, rostral ventrolateral medulla; SFO, subfornical organ. The graphical tool for authors to generate the figure is BioGDP [33]

The major functional circuits of the central autonomic network and sites with reported estrogen receptor expression are summarized in Fig. 1.

### NTS, CVLM, and RVLM pathways in baroreflex and chemoreflex control

The brainstem provides moment-to-moment regulation of sympathetic vasomotor tone through interconnected NTS, CVLM, and RVLM pathways. The NTS receives afferent information from arterial baroreceptors and chemoreceptors and relays it to downstream autonomic regions. During baroreflex activation, excitatory input from the NTS activates inhibitory neurons in the CVLM, which restrain RVLM presympathetic activity and reduce sympathetic outflow. The RVLM subsequently converts the net balance of reflex excitation and inhibition into changes in vasomotor sympathetic activity [3, 14, 15].

Glutamatergic excitation and GABAergic inhibition are key components of this medullary reflex circuit. Excitatory amino acid signaling operates in baroreceptor afferent, NTS, and RVLM pathways, and GABAergic neurons in the CVLM provide inhibitory control within the baroreflex arc. Responses to stimulation, inhibition, or lesioning of these regions differ between spontaneously hypertensive rats (SHR) and Wistar–Kyoto (WKY) controls, indicating altered amino acid neurotransmission in this model [34]. Young SHR exhibit RVLM pressor responses and CVLM depressor responses to L-glutamate that are similar to those in WKY rats, but sympathoinhibitory function of the CVLM is reduced [35]. Thus, early dysfunction may involve weakened network inhibition rather than a generalized increase in local RVLM responsiveness to glutamate.

Altered afferent processing can increase activity in the RVLM and downstream spinal sympathetic pathways. Kainic acid injection into the NTS produced slowly developing hypertension, increased Fos immunoreactivity in the RVLM presympathetic region, and activated spinal sympathetic preganglionic neurons [36]. RVLM NMDA receptors also participate in acute reflex responses. NMDA receptor antagonism attenuated the pressor response to bilateral carotid clamping, and local AP-7 prevented carotid occlusion-induced excitation in a subset of RVLM neurons [16]. RVLM NMDA receptor blockade inhibited pressor and tachycardic responses to carotid body chemoreceptor stimulation, whereas non-NMDA receptor blockade did not produce the same effect [17]. These findings establish the RVLM as an important reflex output region but also show that abnormal NTS or CVLM input can alter RVLM activity without requiring a primary local RVLM defect.

### Circumventricular organs as blood–brain interfaces for neurohumoral and inflammatory signaling

CVOs are specialized periventricular structures characterized by fenestrated capillaries and an incomplete blood–brain barrier. Cardiovascular function-relevant CVOs include the subfornical organ (SFO), organum vasculosum of the lamina terminalis (OVLT), and area postrema (AP). Their vascular properties permit circulating Ang II, cytokines, osmotic signals, and other blood-borne factors to influence central autonomic pathways [2, 3, 18].

The SFO and OVLT primarily transmit circulating neurohumoral signals to hypothalamic regions such as the PVN, while the AP connects with brainstem nuclei such as the RVLM via NTS. Through these neural pathways, CVOs can sense changes in the circulatory environment and further regulate autonomic, neuroendocrine, behavioral, and sympathetic responses [20, 37]. CVOs are not simply relay structures for transmitting signals, but rather important sensory nodes in the central autonomic neural network [2, 3].

### The role of circumventricular organs in hypertension

The SFO provides a clear example of how circulating inflammatory signals can influence cardiovascular regulation. Peripheral administration of TNF-α or IL-1β increases arterial pressure, heart rate, and renal sympathetic nerve activity, despite the limited ability of these cytokines to penetrate an intact blood–brain barrier. SFO lesion markedly attenuates these sympathetic and hemodynamic responses, supporting the SFO as an important forebrain site through which circulating proinflammatory cytokines act [37].

Direct SFO intervention further identifies interactions between inflammatory and renin–angiotensin signaling. Local administration of TNF-α or IL-1β reproduces the sympathetic and hemodynamic responses to systemic cytokine exposure, and these responses are attenuated by local AT1R blockade, ACE inhibition, or COX-2 inhibition. Cytokine exposure also increases inflammatory and excitatory mediators in the SFO and alters ACE, AT1R, and COX-2 expression in the downstream PVN [19]. At the cellular level, TNF-α directly excites SFO neurons and enhances Ang II-induced calcium responses through mechanisms involving AT1R signaling, extracellular calcium, and TTX sensitive voltage-gated sodium channels [38, 39].

Although derived from a systolic heart failure model rather than hypertension, additional evidence supports inflammatory and RAS-related signaling between the SFO and PVN. Selective TNFR1 knockdown in the SFO reduced local TNFR1 expression, decreased inflammatory and RAS-related markers in both the SFO and downstream PVN, attenuated sympathetic activation, and improved cardiac function [21].

Complementing this forebrain pathway, the AP provides a hindbrain interface through which circulating inflammatory signals can influence central autonomic and behavioral responses. Earlier lesion studies showed that AP lesions attenuated TNF-induced anorexia and learned food aversions, supporting a role for the AP in central responses to circulating TNF-related signals [40]. Cardiovascular studies later extended this concept to sympathetic regulation. In renovascular hypertension, AP neurons expressing TNFR1 remain activated, and local TNFR1 blockade substantially lowers blood pressure. TNF-α administration into the AP of normotensive rats increases blood pressure and cardiac and renal sympathetic nerve activity, while local TNFR1 neutralization abolishes these responses. Some TNF-α-responsive AP neurons are retrogradely labeled from the RVLM, supporting a pathway through which inflammatory signals in the AP can influence RVLM activity and sympathetic output [20].

The OVLT also contributes to neurohumoral regulation in hypertension. In the 2K1C renovascular model, OVLT lesion markedly attenuates the hemodynamic, neurogenic, behavioral, neuroendocrine, and renal inflammatory responses associated with hypertension [41]. Together, these studies identify the SFO, AP, and OVLT as active sensory interfaces that convert circulating inflammatory, neurohumoral, and osmotic signals into central autonomic responses. Their effects may reach the RVLM directly, as in the case of the AP, or indirectly through hypothalamic and other network pathways.

### The role of the PVN in hypertension

The PVN is a major hypothalamic nucleus that combines neuroendocrine and autonomic regulatory functions [3]. Classical neuroanatomical studies identified vasopressin and oxytocin neurons within the PVN and demonstrated descending projections to cardiovascular and autonomic regions, including the NTS, dorsal motor nucleus of the vagus, locus coeruleus, and spinal intermediolateral column [22]. These projections provide an anatomical basis through which the PVN can influence cardiovascular function by both neuroendocrine and descending autonomic mechanisms [22].

Early brain region lesion experiments found that disrupting the PVN could attenuate the hypertensive response in some animal models, indicating that the PVN is also involved in the development and maintenance of elevated blood pressure. In SHR, PVN lesions performed at five weeks of age inhibited the subsequent development of hypertension and reduced urinary norepinephrine excretion and the depressor response to ganglionic blockade. Vasopressin antagonism did not lower blood pressure, and urinary vasopressin excretion was not reduced, favoring a predominantly sympathetic rather than circulating vasopressin-dependent mechanism in this model [42]. In Dahl salt-sensitive rats, lesioning the PVN and the adjacent anteromedial hypothalamus could prevent or delay the rise in blood pressure caused by high salt intake; however, no significant effect was observed when the lesion did not include the PVN [43]. Because some lesions extended beyond the PVN, these results implicate the PVN containing the anteromedial hypothalamic region rather than proving that every effect arose exclusively within the PVN.

Further research has revealed the role of the PVN in neuroimmunological regulation. During hypertensive challenge, a distinct vascular arrangement within the PVN permits blood-borne ATP to access the region and activate local microglia through P2Y12 receptors. Downstream C/EBPβ signaling promotes inflammatory responses, including TNF-α and IL-1β expression, and contributes to sympathetic hyperactivity. Disruption of this pathway limits PVN microglial activation and attenuates hypertension development [44].

PVN microglia are not uniformly prohypertensive. Under basal conditions, microglia release PDGFB, which activates neuronal PDGFRα and promotes expression of the potassium channel Kv4.3 in PVN presympathetic neurons. This pathway supports neuronal potassium currents, restrains basal sympathetic tone, and limits hypertensive responses. Its disruption predisposes animals to hypertension, whereas central PDGFB supplementation suppresses pressor responses during hypertensive challenge [45]. Thus, PVN microglia can either promote sympathetic excitation during inflammatory stress or preserve neuronal excitability homeostasis under other conditions.

### Neurogenic hypertension as sympathetic dysregulation at the network level

Neurogenic hypertension is not caused by dysfunction of a single brain region, but rather by the combined dysregulation of multiple interconnected central autonomic neural circuits. Circulatory, neurohumoral, inflammatory, and reflex signals are sensed and processed by regions such as the CVOs, PVN, NTS, and CVLM, influencing sympathetic output through direct or indirect pathways. The RVLM, located at the convergence point of these pathways, integrates upstream inputs and translates them into descending sympathetic vasoconstrictive drives. Persistent dysregulation of this network increases peripheral vascular resistance, promotes renal sodium retention, and maintains elevated blood pressure [2, 46]. Local redox signaling is an important regulator of RVLM neuronal activity and provides a mechanistic link between upstream autonomic inputs and excessive sympathetic output [47, 48].

## The RVLM as a redox-sensitive sympathetic output hub

### Anatomical and neurochemical organization of RVLM presympathetic neurons

The RVLM is a major brainstem region responsible for maintaining sympathetic vasomotor tone and arterial pressure. It contains heterogeneous neuronal populations, including presympathetic neurons that project directly to sympathetic preganglionic neurons in the intermediolateral column of the spinal cord. These bulbospinal neurons provide tonic excitatory drive that contributes to basal sympathetic activity, vascular resistance, and resting arterial pressure. Consistent with this role, pharmacological inhibition of the RVLM markedly reduces sympathetic nerve activity and arterial pressure [23, 24].

The neurochemical identity of RVLM presympathetic neurons is not uniform. Two major bulbospinal populations are recognized: C1 adrenergic neurons, defined by phenylethanolamine N-methyltransferase (PNMT) expression and usually also exhibiting tyrosine hydroxylase (TH) immunoreactivity, and non-C1 presympathetic neurons lacking the C1 adrenergic phenotype. Both populations contribute substantially to descending projections to thoracic sympathetic preganglionic neurons [24–26]. Most barosensitive presympathetic neurons in both populations express vesicular glutamate transporter 2 (VGLUT2) mRNA, supporting glutamate as a principal fast excitatory transmitter [27]. Consistent with this glutamatergic phenotype, C1 neurons have been shown to containVGLUT2 in their central axon terminals and to form monosynaptic glutamatergic connections with parasympatheticpreganglionic neurons in the dorsal motor nucleus of the vagus [49]. Thus, the adrenergic phenotype of C1 neurons does not imply exclusively catecholaminergic transmission, because these neurons can synthesize adrenaline while also using glutamate at central synapses.

Subsets of both C1 and non-C1 barosensitive bulbospinal neurons also express preproenkephalin mRNA, indicating additional peptidergic heterogeneity [50]. Transmitter phenotype and projection identity overlap across neuronal populations and therefore cannot be used to define completely distinct classes of presympathetic neurons [51]. This neurochemical heterogeneity is important when interpreting functional studies because regional variations should be associated with the neuronal population and neurotransmitter phenotype under study and cannot be generalized to all RVLM neurons.

The RVLM also receives convergent inputs from NTS–CVLM reflex pathways, hypothalamic regions, and neurohumoral circuits. Its bulbospinal neurons constitute a major convergence and premotor output pathway that integrates these inputs and converts them into descending sympathetic vasomotor drive.

### Glutamatergic and GABAergic regulation of RVLM sympathetic output

RVLM presympathetic output is governed largely by glutamatergic excitation and GABAergic inhibition, with the CVLM providing a major inhibitory input to RVLM presympathetic neurons [14]. Evidence from SHR indicates that this excitatory-inhibitory balance is altered in hypertension. Spinal glutamate receptor blockade reduces resting blood pressure in SHR but not in WKY rats, supporting an enhanced contribution of excitatory amino acid neurotransmission to blood pressure maintenance in SHR [34]. However, young SHR show RVLM pressor responses and CVLM depressor responses to L-glutamate that are similar to those of WKY rats, but exhibit reduced tonic sympathoinhibitory function of the CVLM [35]. These findings suggest that impaired network-level inhibitory control, not a generalized increase in local RVLM glutamate responsiveness, may emerge early during spontaneous hypertension.

Sex-dependent differences in RVLM responsiveness may also precede cyclic ovarian hormone exposure. In Sprague–Dawley rats, glutamate microinjection into the RVLM produced a greater absolute increase in splanchnic sympathetic nerve activity in four-week-old females than in males, although percentage sympathetic responses and mean arterial pressure responses were similar. This difference persisted at eight and sixteen weeks, was independent of physical activity, and occurred despite lower baseline mean arterial pressure in females [52]. These findings suggest that sex differences in the organization of RVLM sympathoexcitatory pathways may already exist before the onset of the estrous cycle and may be further shaped by reproductive hormones during development.

### Nox isoforms, regulatory subunits, and their localization in the RVLM

Nox enzymes are specialized oxidases whose primary function is the generation of ROS [53]. Members of the Nox family, including Nox1, Nox2, Nox4, and Nox5, have been identified across cardiovascular and neural tissues, and their expression and functional relevance within central autonomic regions differ substantially [47, 48, 53]. These enzymes transfer electrons from NADPH to molecular oxygen, generating superoxide or related ROS [53]. Nox1 and Nox2 require the assembly of membrane-associated and cytosolic regulatory components. The Nox2 complex includes gp91phox/NOX2, p22phox, and cytosolic proteins such as p47phox, whereas Nox1 activation can involve NoxO1 or p47phox-related organizer proteins [54]. Nox4 is generally considered constitutively active and predominantly generates hydrogen peroxide [55]. Nox5 is calcium-dependent [56], but its expression and function in central autonomic regions, particularly the RVLM, have been investigated only sparsely. These differences in activation, ROS products, and subcellular localization may result in distinct redox-signaling effects.

Regional and functional studies demonstrate that redox signaling is active within the RVLM. NADPH oxidase-dependent ROS production is increased in the RVLM in hypertensive models, and local manipulation of oxidative pathways alters sympathetic nerve activity and arterial pressure [47, 48]. RVLM-targeted eNOS overexpression, antioxidant treatment, AT1R blockade, and ARB administration also modify local oxidative stress and sympathetic output [57–59]. However, these functional studies establish the importance of NADPH oxidase-dependent redox signaling in the RVLM but do not by themselves identify the responsible NOX isoforms or their cellular sources.

More specific histological evidence is the strongest for components of the NOX2 complex. Dual-label immunoelectron microscopy demonstrated p47phox immunoreactivity in tyrosine hydroxylase-positive C1 neurons [60]. Ang II-induced ROS production in retrogradely identified bulbospinal RVLM neurons was attenuated by AT1R antagonism and NADPH oxidase inhibition [60]. A subsequent ultrastructural study localized p47phox and p22phox to C1 neuronal dendrites, with labeling detected in both the cytoplasm and plasma membrane [61].

Double-immunofluorescence analysis in rats with acute myocardial infarction showed that approximately 80% of OX1R-immunoreactive RVLM cells co-localized with gp91phox and approximately 30% with p47phox. These findings demonstrate that NOX2-complex components are expressed in OX1R-positive cells within the RVLM. However, the study did not examine their distribution across bulbospinal presympathetic neurons or assess other Nox isoforms [62].

Taken together, the available histological studies support the expression of gp91phox, p22phox, and p47phox in selected RVLM cell populations, particularly C1 neurons and OX1R-positive cells [60–62]. Nevertheless, detection of individual subunits or their co-localization does not by itself demonstrate cell-specific assembly, membrane translocation, or catalytic activity of the complete NOX2 complex. The relative contribution of neuronal, glial, and vascular sources of NOX2-derived ROS therefore remains incompletely resolved.

Evidence for other Nox isoforms within the RVLM remains limited. Although Nox1 and Nox4 have been implicated in oxidative regulation in cardiovascular and other biological contexts [55, 63], their cell-specific expression patterns and functional contributions within the RVLM have not been clearly established. Evidence for Nox5 expression in central autonomic regions, including the RVLM, is currently sparse. Experimental Nox inhibition can reduce oxidative stress and improve cardiovascular outcomes, and peripheral vascular studies demonstrate isoform-specific regulation of Nox1, Nox2, and Nox4 [64–67]. However, these findings provide a broader cardiovascular context rather than direct evidence for defined RVLM expression or function. Thus, current RVLM evidence most strongly supports NOX2-complex involvement, whereas the presence, localization, and functional contribution of other Nox isoforms within the RVLM remain important unresolved questions.

### Ang II–AT1R–Nox–ROS signaling and chronic RAS blockade in the RVLM

Ang II is a major renin–angiotensin system (RAS)-dependent activator of Nox-derived ROS in the RVLM. AT1R activation increases local oxidative stress and sympathetic activity [57, 68, 69]. AT1R blockade attenuates RVLM oxidative stress and sympathoexcitation in hypertensive models [57]. Thus, the Ang II–AT1R–Nox–ROS pathway provides an important mechanistic link between central RAS activation, redox imbalance, and neurogenic blood pressure regulation [68].

Direct RVLM intervention studies have defined this signaling pathway in greater detail. Bilateral Ang II microinjection increased superoxide production and induced AT1R-dependent phosphorylation of p38 MAPK and ERK1/2 [69]. Local administration of the NADPH oxidase inhibitor DPI, antisense oligonucleotides targeting p22phox or p47phox, or the superoxide dismutase mimetic tempol attenuated these molecular responses [69]. DPI, tempol, and the p38 MAPK inhibitor SB203580 also reduced the Ang II-induced pressor response. SB203580 abolished the enhancement of glutamate-sensitive excitatory postsynaptic currents in RVLM neurons [69]. These findings support a local signaling sequence in which Ang II activates AT1R-dependent Nox-derived superoxide, recruits p38 MAPK signaling, enhances excitatory neurotransmission, and produces a sympathetically mediated pressor response.

Ang II microinjection into the RVLM elicited dose-dependent pressor responses in both SHR and Wistar–Kyoto WKY rats, and injection into the CVLM produced depressor responses. Ganglionic blockade abolished the RVLM pressor effect, demonstrating its dependence on sympathetic transmission [70]. Conversely, an Ang II antagonist injected into the RVLM caused depressor and bradycardic responses, whereas the same intervention in the CVLM produced pressor and tachycardic effects; the blood pressure responses were greater in SHR than in WKY rats [70]. These findings identify the RVLM as an Ang II-sensitive sympathoexcitatory site and suggest that endogenous ventrolateral medullary Ang II signaling contributes more strongly to prevailing vasomotor tone in hypertension.

Chronic disease-model evidence indicates that this pathway is not restricted to direct Ang II administration. In adolescent SHR exposed to high-fructose intake, systolic blood pressure and sympathetic vasomotor activity increased more strongly than in normally fed SHR, together with greater RVLM ROS accumulation and lipid peroxidation. AT1R and the Nox2 catalytic subunit gp91phox were upregulated in RVLM neurons; central tempol infusion reduced ROS accumulation and attenuated the exaggerated pressor and sympathetic responses [71].

The central sympathoinhibitory effects of chronic RAS blockade are relevant to this mechanism. In addition to preventing peripheral vascular AT1R-mediated constriction, orally administered AT1R blockers can reduce sympathetic nerve activity by attenuating AT1R-dependent oxidative stress in central cardiovascular regions, including the RVLM [72]. In obesity-prone hypertensive rats, telmisartan reduced systolic blood pressure, RVLM oxidative stress, and sympathetic nervous system activity and produced greater sympathoinhibition than losartan despite a similar reduction in blood pressure [58].

Systemic RAS blockade and direct RVLM interventions provide complementary but distinct evidence. Because orally administered RAS inhibitors act at multiple peripheral and central sites, their effects on sympathetic activity cannot be attributed solely to inhibition of RVLM Nox signaling [58, 72]. This attribution is further complicated by peripheral vascular mechanisms. Comparative studies across hypertensive models showed that the nifedipine-sensitive blood pressure component is proportional to the prevailing vasoconstrictor activity generated by both the sympathetic nervous system and the RAS [73]. Taken together, the available findings indicate that RVLM AT1R–Nox–ROS signaling contributes to the development and persistence of sympathetic activation. The magnitude of this contribution is likely to vary with the hypertensive model, disease duration, drug access to the central nervous system, and the strength of upstream neural and neurohumoral inputs [57].

Comparable Ang II–Nox–ROS coupling has been demonstrated in other central autonomic nuclei. In the PVN, losartan, tempol, or apocynin attenuated Ang II-induced sympathetic excitation in obesity-related hypertension [74]. This finding supports a distributed central RAS–redox mechanism, with RVLM-specific confirmation requiring local measurements [74]. Human RVLM Nox activation has not yet been measured directly; its translational relevance therefore relies on convergent evidence from RAS dependence, sympathetic overactivity, systemic oxidative stress, and animal studies [68].

### Interacting redox mechanisms: NO, mitochondrial dysfunction, and neuroinflammation

Nox-derived ROS interact with nitric oxide (NO) signaling, mitochondrial redox homeostasis, neuroinflammation, and glial regulation to shape RVLM sympathetic output [14, 75]. These pathways may either restrain oxidative signaling or amplify local redox imbalance and sympathoexcitation.

NO–GABA signaling provides an important counter-regulatory mechanism within the RVLM. eNOS overexpression studies indicate that locally generated NO suppresses sympathetic activity by enhancing GABAergic signaling, and oxidative stress reduces NO bioavailability and weakens this inhibitory control [57]. In stroke-prone spontaneously hypertensive rats (SHRSP), superoxide scavenging with tempol increased RVLM GABA release and reduced arterial pressure, heart rate, and renal sympathetic nerve activity. Conversely, NOS inhibition decreased GABA release and produced sympathoexcitation, and these responses were attenuated by GABA receptor blockade. Excessive superoxide can increase RVLM sympathetic output partly by impairing NO-dependent GABAergic inhibition [59].

Mitochondrial dysfunction and neuroinflammation form an additional redox-amplifying pathway. Microglia-mediated neuroinflammation within central autonomic nuclei contributes to cardiovascular disease and hypertension [76]. In the RVLM, microglia-derived TNF-α disrupts neuronal AMPK–Sirt3 signaling, induces mitochondrial dysfunction, and promotes stress-induced hypertension [77]. This mechanism suggests that immune activation may disrupt mitochondrial redox balance in RVLM neurons. However, the sequence of these events and the respective contributions of Nox-derived and mitochondrial ROS remain unclear.

Glial dysfunction may further connect redox imbalance with excitatory neurotransmission. Astrocytes regulate extracellular glutamate through glutamate uptake, and impaired astrocytic transport increases extracellular glutamate, enhances neuronal excitability, and induces hypertensive responses [78]. Astrocyte-derived ROS, endoplasmic reticulum stress, and central aldosterone signaling may also increase the local oxidative burden [79]. The contribution of these mechanisms varies with disease context: salt-sensitive hypertension and age-related hypertension susceptibility are associated with differential RVLM oxidative stress responses [80, 81]. Heart-failure studies further support reciprocal interactions among central inflammation, RAS activation, oxidative stress, and sympathetic excitation, although they provide broader central autonomic network evidence rather than direct proof of an identical RVLM-specific mechanism in hypertension [82].

Endogenous antioxidant and adaptive pathways help limit these pro-oxidant processes. SIRT1–FOXO1 activation reduces oxidative stress and modulates RVLM neuronal activity [83]. β-Arrestin1 also suppresses oxidative stress through the Nrf2 antioxidant pathway [84]. Conversely, miR-22-3p promotes hypertension by inhibiting β-arrestin1 signaling in the RVLM [85]. RVLM redox balance is determined by the interaction between ROS-generating pathways and endogenous antioxidant defenses.

### Functional consequences of Nox-derived ROS for RVLM neuronal excitability and sympathetic output

Nox-derived ROS can translate upstream neurohumoral, metabolic, and inflammatory signals into changes in RVLM neuronal activity. Compartmentalized ROS regulate ion-channel activity, intracellular signaling, synaptic transmission, and neuronal plasticity, thereby altering the excitatory–inhibitory balance that determines presympathetic output [47, 86, 87].

At the membrane level, oxidative modification of redox-sensitive voltage-gated potassium channels can alter membrane conductance, firing thresholds, and neuronal discharge, thereby favoring enhanced excitability under pro-oxidant conditions [88]. In retrogradely identified bulbospinal RVLM neurons, Ang II produced comparable NADPH oxidase-dependent ROS responses in males and females but enhanced L-type Ca²⁺ currents more strongly in female neurons. This pattern indicates that the sex difference may occur downstream of ROS generation, at the level of redox-sensitive regulation of membrane excitability [60].

Nox-derived ROS also influence excitatory synaptic transmission. As detailed above, Ang II–AT1R–Nox signaling enhances glutamate-sensitive excitatory postsynaptic currents through p38 MAPK, linking local superoxide production to excitatory synaptic output and pressor responses [69]. These findings establish a functional connection among Nox-derived superoxide, intracellular kinase signaling, glutamatergic excitation, and cardiovascular output without requiring nonspecific excitation of all RVLM neurons.

Excess ROS can simultaneously weaken inhibitory control. Oxidative inactivation of NO or NOS uncoupling reduces NO bioavailability, shifting RVLM signaling toward a sympathoexcitatory state [89, 90]. Nitric oxide derived from eNOS facilitates GABAergic inhibition in the RVLM, but excess superoxide reduces NO-dependent GABA release and promotes sympathetic activation [57, 59]. Redox imbalance may therefore increase RVLM output by enhancing glutamatergic excitation while weakening inhibitory control.

In addition to these relatively rapid membrane and synaptic effects, ROS regulate pathways involved in gene transcription, cellular metabolism, receptor trafficking, and synaptic plasticity, potentially producing more sustained changes in sympathetic regulation [86, 87]. In a slow-pressor Ang II model, hypertension in male mice was accompanied by increased plasma-membrane GluN1 in CRF1-containing RVLM dendrites, these changes were not observed in females [91].

The net functional consequence of these membrane, synaptic, and intracellular changes is increased activity of RVLM presympathetic neurons and greater descending sympathetic drive to spinal sympathetic preganglionic neurons. Local suppression of NADPH oxidase components, superoxide, or downstream redox-sensitive signaling attenuates pressor responses [69]. In hypertensive models, antioxidant treatment also reduces sympathetic nerve activity and arterial pressure, partly by preserving NO-dependent GABAergic inhibition [14, 48, 59].

The RVLM contains diverse neuronal and glial populations. Redox-sensitive changes have been reported mainly in bulbospinal neurons, C1 neurons, and selected receptor-expressing cells [60]. Oxidative signaling in this region may alter presympathetic output through specific cell populations and neural circuits, without affecting the entire RVLM uniformly.

## Pathophysiological significance of RVLM oxidative stress in hypertension

### Convergence of Nox-centered redox mechanisms in the RVLM

RVLM oxidative stress develops through the combined influence of neurohumoral, metabolic, inflammatory, and cellular signals. Nox enzymes are an important regulated source of ROS in this region, but mitochondrial dysfunction, NOS uncoupling, impaired antioxidant defenses, and glial activation also contribute to the local redox environment [14, 47, 79, 80, 90]. These processes can reinforce one another, allowing a disturbance initiated through one pathway to produce a broader and more persistent redox imbalance.

This integrated redox disturbance can increase RVLM presympathetic output by strengthening excitatory signaling and weakening inhibitory regulation. Nox-derived superoxide can reduce NO availability and impair NO-dependent GABAergic control, while mitochondrial and glial abnormalities may further sustain neuronal excitation [57, 59, 76–78]. Endogenous antioxidant pathways limit these effects but may become insufficient during hypertension [83–85]. RVLM oxidative stress is therefore best viewed as an interacting pathological network whose contribution depends on the disease state and the strength of the upstream signals reaching the region.

### Disease-state dependence and evidence boundaries: amplification and maintenance of established hypertension

Acute and subacute studies demonstrate the pressor capacity of RVLM-related pathways, but they do not establish that RVLM oxidative stress independently initiates chronic hypertension. Local Ang II administration produces AT1R–Nox–ROS-dependent pressor responses, while experiments involving RVLM NMDA receptors and altered NTS input show that reflex and excitatory pathways can increase RVLM activity and sympathetic output [16, 17, 36, 69, 70]. Evidence for a primary role in disease initiation, however, requires continuous longitudinal studies that distinguish an initiating mechanism from a response recruited after blood pressure has already begun to rise.

Evidence from established hypertensive models more strongly supports a disease-state-dependent role in the amplification and maintenance of sympathetic activation. Inhibition of oxidative or NADPH oxidase-dependent signaling within the RVLM generally produces greater sympathoinhibitory and depressor effects in hypertensive animals than in normotensive controls [47, 48, 59]. Similarly, endogenous ventrolateral medullary Ang II signaling appears to make a greater contribution to prevailing vasomotor tone in SHR than in WKY rats [70]. These findings indicate that RVLM redox mechanisms become more functionally important once hypertension and sympathetic overactivity are established.

Metabolic and dietary challenges further enhance the contribution of these pathways. High-fructose intake, high-salt exposure, and metabolic syndrome-related NOS uncoupling are associated with increased RVLM oxidative signaling and greater sympathetic or pressor responses [71, 80, 90]. Findings from chronic AT1R blockade are also consistent with a contribution from central redox mechanisms [58, 72]. However, because systemic RAS inhibitors act at multiple central and peripheral sites, their cardiovascular and sympathoinhibitory effects cannot be attributed specifically to inhibition of RVLM Nox signaling. Systemic NO-deficient models illustrate a similar attribution problem: in chronic L-NAME hypertension, blood pressure maintenance depended more strongly on sympathetic activity than on the RAS, and residual iNOS-derived NO appeared to provide a compensatory but insufficient vasodilator influence [92].

Most available evidence is derived from animal models, and direct involvement of RVLM Nox signaling in human essential hypertension remains unconfirmed, largely because it is difficult to demonstrate due to ethical constraints [68]. In addition, the redox state of RVLM presympathetic neurons is shaped by inputs from the wider central autonomic network. Reflex signals transmitted through the NTS–CVLM pathway and neurohumoral or inflammatory signals arising from the CVOs and PVN may alter RVLM neuronal activity and local oxidative signaling [2, 3, 20, 36, 42]. Estrogen-sensitive regulation in these upstream regions may therefore modify the signals reaching the RVLM and shape its local redox environment.

## Estrogen signaling in upstream central autonomic regions: the PVN and circumventricular organs

### Basic estrogen signaling framework: ligands, receptor subtypes, and central access

Estrogen exerts diverse effects in the central nervous system through multiple receptor-mediated signaling pathways [93]. The term estrogen encompasses several estrogenic compounds, with 17β-estradiol (E2) representing the predominant biologically active ovarian estrogen. Estrogenic ligands and metabolites differ in receptor affinity, metabolism, tissue access, and downstream signaling [93].

Three major estrogen receptors are relevant to central autonomic regulation: estrogen receptor alpha (ERα), estrogen receptor beta (ERβ), and the G protein-coupled estrogen receptor (GPER) [93]. These receptors are distributed across hypothalamic and brainstem autonomic regions, but their relative abundance, cellular localization, and functional consequences differ among nuclei and neuronal or glial populations [29, 93, 94].

E2 is a lipophilic steroid and can access the CNS more readily than many hydrophilic signaling molecules [93]. Circulating concentration alone does not determine the estrogenic signal received by a central autonomic nucleus. Plasma binding proteins, local steroid metabolism, receptor expression, administration route, and treatment duration all influence effective central exposure [93]. Accordingly, estrogenic effects should be interpreted according to ligand identity, receptor subtype, dose, exposure duration, age, ovarian status, administration route, and disease context [8]. Endogenous ovarian signaling, systemic replacement, receptor-selective stimulation, and local central administration therefore represent distinct experimental conditions.

Anatomical access also differs among autonomic regions. Circumventricular organs such as the SFO, OVLT, and AP have fenestrated capillaries and an incomplete blood–brain barrier, allowing them to detect circulating Ang II, cytokines, and osmotic or metabolic signals [2, 3, 18]. The PVN is not conventionally classified as a circumventricular organ, but its vascular microenvironment may permit selected blood-borne signals to influence local cells during hypertension. For example, blood-borne ATP can enter the PVN during hypertensive challenge and activate local microglia [44].

Central estrogenic actions must therefore be interpreted using a region-specific framework. Ligand identity, receptor subtype, cellular phenotype, endocrine state, vascular accessibility, and disease background jointly determine the response; circulating E2 levels alone cannot predict the direction or magnitude of signaling in the PVN, CVOs, or RVLM.

### Estrogen-sensitive inflammatory and synaptic signaling in the PVN

The PVN is an important hypothalamic sympathoregulatory nucleus implicated in experimental hypertension [2, 42]. ERβ-containing neuronal populations are present in the PVN, and their distribution varies with sex, age, and neuronal phenotype [29]. In female mouse models, PVN ERβ signaling has been directly linked to inflammatory-redox regulation during slow-pressor Ang II exposure [95].

Fourteen-day slow-pressor Ang II infusion alone did not significantly increase blood pressure or alter PVN TNF-α, TNFR1 expression, or TNFR1 localization. Addition of the selective ERβ antagonist PHTPP induced hypertension, increased PVN TNF-α, and enhanced p47phox phosphorylation at Ser304. Local TNFR1 silencing attenuated the hypertensive response. In isolated PVN–spinal projection neurons, ERβ blockade amplified TNF-α-induced ROS production, and this effect was reduced by TNFR1 deletion or NOX2 inhibition with gp91ds [95].

In ERβ-EGFP female mice subjected to accelerated ovarian failure (AOF), slow-pressor Ang II increased systolic blood pressure during peri-AOF and post-AOF, but not in oil-treated controls or pre-AOF VCD-treated females [96]. During peri-AOF, hypertension was accompanied by redistribution of the obligatory NMDA receptor subunit GluN1 toward the plasma membrane in ERβ-containing PVN dendrites [96]. During post-AOF, total GluN1 density, dendritic size, and ROS production increased without the same pattern of surface redistribution. These findings indicate stage-dependent synaptic and redox remodeling in the PVN during ovarian failure [96].

Additional studies indicate that estrogen-sensitive PVN regulation may involve other membrane mechanisms. Estrogen replacement modulates voltage-gated potassium channels in rat presympathetic PVN neurons [97]. Postmenopausal hypertension in mice has been associated with increased plasma-membrane-affiliated AMPA GluA1 in ERβ-containing PVN neurons [98]. Because these studies differ in species, model, receptor system, and ion channel, they should be regarded as complementary evidence rather than components of a single established pathway.

The PVN neuroimmune environment is also state dependent. Blood-borne ATP can activate microglial P2Y12–C/EBPβ signaling and promote inflammatory and sympathetic responses during hypertension [44]. Under other conditions, microglial PDGFB–neuronal PDGFRα–Kv4.3 signaling supports potassium currents and restrains presympathetic neuronal activity [45]. These findings demonstrate opposing microglial influences within the PVN, but current evidence does not show that ERβ directly controls either pathway.

### Estrogen-sensitive mechanisms in circumventricular organs

Circumventricular organs provide interfaces through which circulating neurohumoral and inflammatory signals can influence central autonomic regulation. Estrogen receptor expression in these regions may modify their sensitivity to Ang II, cytokines, and other circulating mediators, although the strength of direct receptor-dependent evidence differs substantially among individual CVOs [94, 99].

Genetic knockdown of ERα in the SFO augmented Ang II-induced hypertension and central sympathetic pressor responses in female mice, supporting a role for SFO ERα in limiting Ang II-dependent hypertension [28]. The location of the SFO at the blood-brain interface enables estrogen receptor signaling to modify central responses to circulating Ang II [2, 3, 18].

This receptor-specific finding is consistent with the established interaction between inflammatory and renin–angiotensin signaling in the SFO. TNF-α directly excites SFO neurons and enhances Ang II-induced calcium responses through mechanisms involving AT1R signaling, extracellular calcium, and TTX-sensitive voltage-gated sodium channels [38, 39]. Local administration of TNF-α or IL-1β also increases arterial pressure, heart rate, and renal sympathetic nerve activity and alters inflammatory signaling and pathways involving the renin–angiotensin system and COX-2 within the SFO and downstream PVN [19]. These studies provide a mechanistic context in which ERα could modify the integration of Ang II and inflammatory signals. However, direct involvement of ERα in these cytokine-dependent responses has not yet been demonstrated.

Evidence for estrogen receptor-dependent regulation in the area postrema and OVLT is more limited. TNFR1 signaling in the AP contributes to renovascular hypertension, and some TNF-α-responsive AP neurons project to the RVLM, providing a route through which circulating inflammatory signals can influence sympathetic output [20]. The OVLT also contributes to the neurohumoral, behavioral, and renal responses associated with 2K1C renovascular hypertension, as lesioning this region attenuates the rise in arterial pressure, neurogenic pressor activity, vasopressin release, water intake, and renal inflammation [41].

Among the CVOs, receptor-dependent evidence is therefore strongest for ERα-mediated modulation of Ang II sensitivity in the SFO [28]. The inflammatory and neurohumoral functions of the AP and OVLT provide plausible settings for estrogen-sensitive regulation, but direct functional evidence remains sparse. Estrogenic signaling in these upstream sensory regions may modify the input ultimately reaching RVLM presympathetic neurons, although such effects do not establish that the same receptor-dependent pathways operate locally within the RVLM.

## Estrogen regulation of RVLM redox signaling and sympathetic output

### Estrogen receptors and direct estrogenic actions in the RVLM

ERα and ERβ are both present in the RVLM C1 region but show distinct distributions. ERβ immunoreactivity is detected mainly in tyrosine hydroxylase-positive neuronal somata and dendrites, and ERα is more prominent in afferent profiles contacting tyrosine hydroxylase-positive dendrites [9].

Electrophysiological findings provide complementary functional evidence. In retrogradely identified bulbospinal RVLM neurons, 17β-estradiol and an agonist selective for ERβ rapidly inhibited L-type Ca²⁺ currents [9]. The rapid time course supports a nongenomic component rather than an effect requiring prolonged transcriptional regulation [100]. Consistent with these cellular findings, local administration of 17β-estradiol or an ERβ agonist into the RVLM acutely reduced arterial pressure and sympathetic vasomotor tone through a nontranscriptional mechanism involving nitric oxide [100].

In male rats with Goldblatt 2 K-1 C hypertension, RVLM expression of ERα and GPER increased, but ERβ expression remained unchanged. Local GPER activation increased mean arterial pressure and renal sympathetic nerve activity. Local 17β-estradiol produced depressor and sympathoinhibitory responses. Losartan reduced the hypertension-associated increases in estrogen receptor expression [11].

To facilitate comparison across central autonomic regions, Table 1 summarizes the available evidence on estrogen receptor subtypes and their hypertension-related mechanisms in the PVN, RVLM, and CVOs.

**Table 1:** Estrogen receptor subtypes and hypertension-related mechanisms in central autonomic regions

Central region	Receptor subtype	Hypertension-related mechanism	References
PVN	ERβ	Restrains TNF-α/TNFR1/NOX2-related ROS signaling. Ovarian failure and postmenopausal hypertension are associated with GluN1 and GluA1 remodeling, respectively, in ERβ-containing neurons, potentially enhancing excitatory signaling	[95, 96, 98, 101
RVLM	ERβ	Rapidly inhibits L-type Ca²⁺ currents and reduces sympathetic vasomotor tone and blood pressure through a rapid NO-dependent, nontranscriptional mechanism	[9, 100]
RVLM	ERα	More prominent in afferent profiles contacting TH-positive C1 dendrites; expression increases in male Goldblatt hypertensive rats, but its receptor-specific effect on blood pressure remains unresolved	[9, 11]
RVLM	GPER	Local activation increases mean arterial pressure and renal sympathetic nerve activity in Goldblatt hypertensive rats	[11]
SFO	ERα	Limits Ang II-induced hypertension and central sympathetic pressor responsiveness; ERα knockdown augments both responses	[28]
AP and OVLT	Not established	These regions participate in inflammatory and neurohumoral regulation of hypertension, but direct estrogen receptor-dependent mechanisms have not been established	[20, 41]

AP, area postrema; ERα, estrogen receptor alpha; ERβ, estrogen receptor beta; GPER, G protein-coupled estrogen receptor; NOX2, NADPH oxidase 2; OVLT, organum vasculosum of the lamina terminalis; PVN, paraventricular nucleus of the hypothalamus; ROS, reactive oxygen species; RVLM, rostral ventrolateral medulla; SFO, subfornical organ; TNF-α, tumor necrosis factor alpha; TNFR1, tumor necrosis factor receptor 1

### Estrogen regulation of Nox and redox signaling in the RVLM across experimental contexts

Estrogenic regulation of RVLM redox balance varies across Nox isoforms and experimental conditions. Nox1 and Nox2 are multisubunit oxidases commonly associated with superoxide-dependent signaling. Nox4 is often constitutively active and may generate different ROS profiles depending on the cellular context [55, 56]. Changes in a single Nox isoform, antioxidant protein, or ROS endpoint therefore reflect one component of the redox system rather than a uniform response of the entire NADPH oxidase family.

Evidence for an antioxidative effect is the strongest in models of ovarian hormone loss followed by replacement. In ovariectomized rats, water-soluble 17β-estradiol administered at 30 µg/kg/day for four weeks prevented increases in blood pressure, heart rate, renal sympathetic nerve activity, and urinary norepinephrine. Estradiol replacement also reduced RVLM ROS and attenuated both the increase in NOX4 and the decrease in SOD1 associated with ovariectomy [10]. These findings indicate improved RVLM redox balance after estradiol replacement.

Prolonged estradiol exposure in animals with intact ovarian function produced a different pattern. Intact cycling female Sprague-Dawley rats receiving a sustained-release estradiol pellet delivering 20 ng/day for 90 days developed increased mean arterial pressure, heart rate, and RVLM superoxide. Resveratrol attenuated these changes [12]. In a related paradigm, chronic estradiol exposure increased RVLM Nox1 and Nox2 mRNA in young females. Middle-aged females showed stronger induction of inflammatory genes and altered antioxidant regulation [13].

These findings arise from biologically distinct endocrine conditions. Estradiol replacement after ovariectomy restores hormonal signaling after ovarian hormone loss. Prolonged exposure in intact cycling animals adds estradiol to an existing endogenous hormonal background [10, 12, 13]. The reported increases in Nox1 and Nox2 mRNA indicate transcriptional regulation and should not be equated with changes in protein abundance, oxidase complex assembly, catalytic activity, or isoform-specific ROS production.

Additional RVLM evidence links ovarian hormone loss to sympathetic signaling that is sensitive to redox changes without identifying a specific Nox isoform. Sympathetic hyperactivity after ovariectomy has been associated with regulation of β-arrestin1 within the RVLM that depends on cFos [102]. This pathway may provide a non-isoform-specific connection between ovarian hormone status and RVLM redox regulation.

### Context-dependent estrogenic regulation of RVLM redox signaling and sympathetic output

The apparently divergent effects of estrogenic signaling in the RVLM reflect differences in endocrine state, ligand exposure, receptor subtype, age, hypertensive background, and the molecular endpoint being measured.

Endocrine context strongly influences the response to estradiol. Replacement after ovariectomy restores hormonal signaling lost with ovarian removal, while prolonged treatment in intact cycling animals adds estradiol to an existing endogenous hormonal background. The regimen using water-soluble estradiol for four weeks and the regimen using a pellet that released estradiol continuously for 90 days differed in formulation and treatment duration [10, 12]. Because neither study directly measured estradiol concentrations within the RVLM, the nominal daily doses cannot be used to compare local estradiol exposure.

Receptor subtype and administration site provide another level of variation. Acute local activation by 17β-estradiol or selective activation of ERβ can inhibit L-type Ca²⁺ currents and reduce sympathetic vasomotor tone [9, 100]. By contrast, GPER activation in the RVLM of male Goldblatt hypertensive rats increased mean arterial pressure and renal sympathetic nerve activity [11]. These results show that 17β-estradiol exposure, selective receptor activation, and receptor remodeling during disease represent distinct experimental manipulations. Receptor effects should therefore be interpreted within the specific models examined rather than being assigned universal protective or harmful roles.

The redox endpoints also differed across studies. The ovariectomy study measured RVLM ROS, NOX4, and SOD1 [10]. The chronic-exposure studies assessed superoxide, Nox1 and Nox2 transcripts, inflammatory genes, and physiological responses [12, 13]. The NOX4 result informs NOX4-specific regulation but not Nox1 or Nox2 activity, while Nox1 or Nox2 mRNA elevation alone does not quantify enzymatic activity. Findings showing no change also require context. For example, ERβ expression was unchanged in the Goldblatt model despite remodeling of ERα and GPER [11]. Ang II also produced comparable NADPH oxidase-dependent ROS in male and female RVLM neurons despite different effects on L-type Ca²⁺ currents [60].

Studies in other central nervous system regions indicate that estrogen receptors can influence inflammation, oxidative injury, and neuronal excitation. Signaling mediated by ERα has been associated with neuroprotective and anti-inflammatory effects in neural tissues [103], and estrogen can attenuate neuronal excitation mediated by cannabinoid receptors in experimental pressor models [104]. Female resistance to hypertension induced by Ang II has also been associated with reduced ROS production in hypothalamic neurons [101].

Age and ligand profile may also shape RVLM responses to estrogenic signaling. Young and middle-aged animals show different inflammatory and antioxidant responses to chronic estradiol exposure [13]. Goldblatt hypertension is accompanied by RVLM estrogen receptor remodeling, indicating that a hypertensive background can further modify these responses [11]. Most direct RVLM studies have focused on 17β-estradiol. The effects of estrone, estriol, catechol estrogens, and methoxy estrogen metabolites on RVLM Nox signaling and sympathetic output have received little direct study.

Collectively, sex differences in RVLM sympathetic regulation involve receptor expression, redox-sensitive membrane excitability, glutamatergic responsiveness, and synaptic plasticity [52, 60, 91]. Some functional differences are detectable before stable cyclic ovarian hormone exposure, including greater absolute sympathoexcitatory responses to RVLM glutamate stimulation in young female rats [52]. Estrogen contributes to these differences, but the direction and magnitude of its effects vary with endocrine state, receptor subtype, exposure pattern, age, and disease model [9–13, 100]. Estrogen may therefore modify pre-existing sex-dependent neural organization rather than acting as a uniformly protective or prooxidant regulator. Furthermore, similar Ang II-induced ROS responses in male and female neurons can produce different electrophysiological effects, indicating that sex differences may arise downstream of ROS generation [60].

Taken together, current evidence suggests that estrogenic modulation of RVLM redox signaling and sympathetic regulation depends on endocrine status, receptor subtype, exposure paradigm, age, and hypertensive background. However, the cellular distribution of ERα, ERβ, GPER, and individual Nox isoforms, as well as their functional interactions within the RVLM, remain insufficiently characterized. Clarifying these relationships will be important for determining how experimental findings relate to changes in autonomic and blood pressure regulation during menopause and MHT.

To distinguish direct male–female comparisons from manipulations of ovarian hormone status or E2 exposure, Table 2 summarizes the available experimental evidence linking sex or endocrine state to RVLM oxidative stress and blood pressure or autonomic outcomes across different experimental paradigms.

**Fig. 2:**
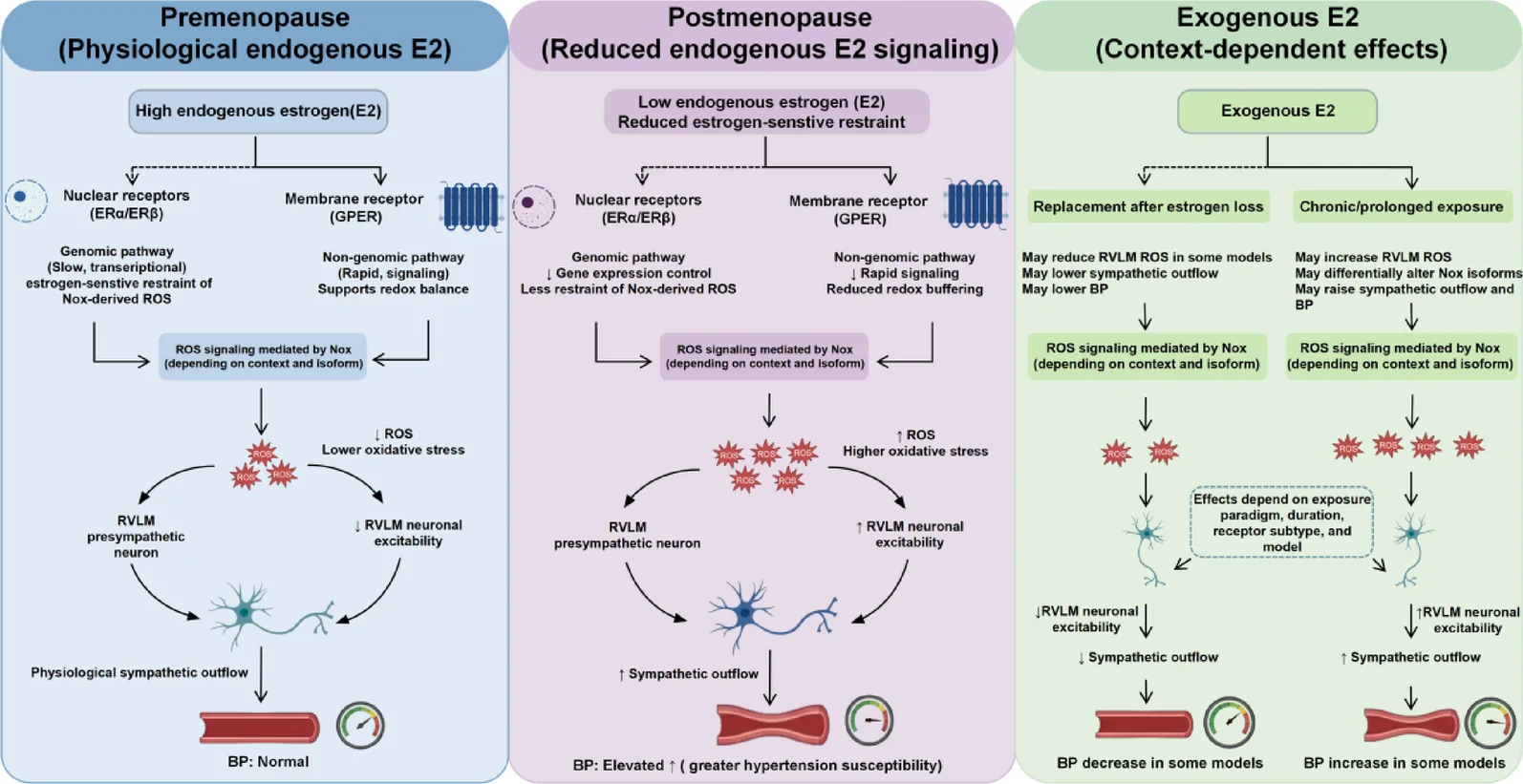
**Context-dependent effects of endogenous and exogenous estrogen on RVLM redox signaling and blood pressure. **Physiological endogenous estradiol (E2) signaling may restrain Nox-derived ROS production and RVLM neuronal excitability, whereas reduced E2 signaling after menopause may favor oxidative stress and sympathetic activation. Exogenous E2 produces variable effects: replacement after estrogen loss may reduce RVLM ROS, whereas chronic exposure may increase ROS and differentially regulate Nox isoforms. These responses depend on the exposure pattern, duration, receptor subtype, and experimental model [9-15, 99, 101, 105-110]. ERα, estrogen receptor alpha; ERβ, estrogen receptor beta; GPER, G protein-coupled estrogen receptor; RVLM, rostral ventrolateral medulla. The graphical tool for authors to generate the figure is BioGDP [33]

Figure 2 illustrates how endogenous estrogen status and different patterns of exogenous E2 exposure may differentially influence RVLM redox signaling, neuronal excitability, sympathetic outflow, and blood pressure.

**Table 2:** Experimental evidence for sex- and ovarian hormone-related variation in RVLM oxidative stress and blood pressure regulation

Experimental model or paradigm	Sex or endocrine comparison	RVLM oxidative stress and related mechanism	Blood pressure or autonomic outcome	References
Ang II stimulation of acutely dissociated, retrogradely identified bulbospinal RVLM neurons	Male versus female rats	Ang II induced comparable AT1R- and NADPH oxidase-dependent ROS production in male and female neurons but enhanced L-type Ca²⁺ currents more strongly in female neurons	Blood pressure and sympathetic nerve activity were not directly measured. The findings suggest that the observed sex difference occurred downstream of ROS generation, at the level of redox-sensitive ion channel excitability	[60]
Ovariectomy model with E2 replacement	Sham-operated, ovariectomized, and ovariectomized plus E2-treated female rats; water-soluble E2 at 30 µg/kg/day for 4 weeks	Ovariectomy increased RVLM ROS and NOX4 expression and reduced SOD1 expression. E2 replacement attenuated these changes and improved local redox balance	Ovariectomy increased blood pressure, heart rate, renal sympathetic nerve activity, and urinary norepinephrine. E2 replacement prevented or attenuated these increases	[10]
Chronic E2 exposure in intact cycling female rats with or without resveratrol	Intact cycling female rats receiving vehicle, sustained-release E2 at 20 ng/day for 90 days, or E2 plus resveratrol	Chronic E2 exposure increased RVLM superoxide production. Resveratrol attenuated the increase in superoxide, supporting an oxidative-stress-dependent component in this paradigm	Chronic E2 exposure increased mean arterial pressure and heart rate, whereas resveratrol attenuated these cardiovascular responses	[12]
Chronic E2 exposure in young and middle-aged female rats	Young and middle-aged female rats receiving vehicle or prolonged E2 treatment	Chronic E2 exposure increased RVLM Nox1 and Nox2 mRNA in young rats, whereas middle-aged rats showed stronger inflammatory and altered antioxidant gene responses. These changes do not establish increased Nox activity or ROS production	Chronic E2 exposure increased mean arterial pressure, with age-dependent RVLM molecular responses. Transcript changes were not shown to cause the blood pressure increase	[13]
Ovarian hormone manipulation combined with local RVLM CB1R activation	Proestrus sham-operated, ovariectomized, and ovariectomized plus E2-treated female rats	Intra-RVLM CB1R activation produced greater ROS and Akt–nNOS signaling responses after ovariectomy. These responses were attenuated in proestrus and E2-replaced rats	Local CB1R activation produced greater pressor and tachycardic responses after ovariectomy, whereas E2 replacement attenuated the cardiovascular responses	[104]

Ang II, angiotensin II; AT1R, angiotensin II type 1 receptor; CB1R, cannabinoid receptor 1; E2, 17β-estradiol; NOX4, NADPH oxidase 4; Nox, NADPH oxidase; nNOS, neuronal nitric oxide synthase; ROS, reactive oxygen species; RVLM, rostral ventrolateral medulla; SOD1, superoxide dismutase 1

## Translational perspectives: menopause, aging, and human relevance

### Menopause and changes in estrogen-sensitive cardiovascular regulation

Menopause is associated with increased hypertension risk, but this transition reflects the combined influences of declining ovarian hormones, aging, adiposity, metabolic dysfunction, vascular remodeling, and altered autonomic regulation [8]. In a cross-sectional study of 313 previously healthy adults, postmenopausal women had higher systolic blood pressure than premenopausal women, but menopausal status was no longer an independent predictor after adjustment for age and body mass index (BMI) [105]. Estradiol decline alone therefore does not explain the higher blood pressure observed after menopause.

Longitudinal data from 3302 participants in the Study of Women’s Health Across the Nation further demonstrated substantial heterogeneity. In selected groups with initially low systolic pressure, pulse pressure, or mean arterial pressure, the rate of increase accelerated around the final menstrual period, whereas diastolic pressure did not show the same pattern. Vasomotor symptoms were associated with higher blood pressure, but circulating estradiol did not independently predict blood pressure trajectory or level [106]. Vascular changes accompany these hemodynamic patterns. In 52,891 women from the UK Biobank, postmenopausal status remained associated with greater arterial stiffness after multivariable adjustment [111]. Perimenopausal symptoms were also more frequent among women with hypertension in a South China cohort, although the cross-sectional design did not establish causality [112].

Reducedestrogen-sensitive endothelial nitric oxide signaling, increased inflammation and oxidative stress, and changes in vascular collagen and elastin may contribute to arterial stiffening. Greater stiffness can increase systolic load and reduce deformation of carotid and aortic barosensory regions, thereby modifying reflex buffering of blood pressure. Hypertensive vascular remodeling may also involve changes in vascular smooth muscle cell phenotype, Ca²⁺ handling, and excitation-transcription coupling, although the contribution of a shift from the contractile to the proliferative phenotype remains uncertain [113].

Alongside these peripheral vascular changes, experimental and clinical evidence suggests that ovarian hormone status can influence central redox and autonomic regulation [10, 107–109]. Ovarian hormone loss followed by estradiol replacement and prolonged estradiol exposure in animals with intact ovarian function produce different RVLM redox responses [10, 12, 13]. The relevance of these findings to postmenopausal women remains uncertain because Nox activation has not been demonstrated directly in the human RVLM.

Importantly, experimental findings do not support a uniformly protective effect of estrogen. Piťha et al. showed that the consequences of ovariectomy and estrogen supplementation vary with strain, age, salt exposure, hypertensive background, estrogen formulation and dose, and the interval between ovariectomy and treatment [110]. In adult female SHR, ovariectomy can increase sympathetic tone and brainstem AT1R expression, whereas its pressor effect is smaller, delayed, or absent in other experimental settings; estrogen replacement likewise improves blood pressure, oxidative stress, or vascular function only in selected models. Treatment timing may also be important, as E2 initiated one or four months after ovariectomy restored several vascular responses, whereas treatment delayed for eight months did not [110]. Ovariectomy is therefore an informative but incomplete approximation of human menopause.

Menopause is best understood as a heterogeneous cardiovascular transition that alters vascular and autonomic sensitivity to aging, metabolic stress, inflammation, and redox imbalance rather than as an isolated state of estrogen deficiency.

**Fig. 3:**
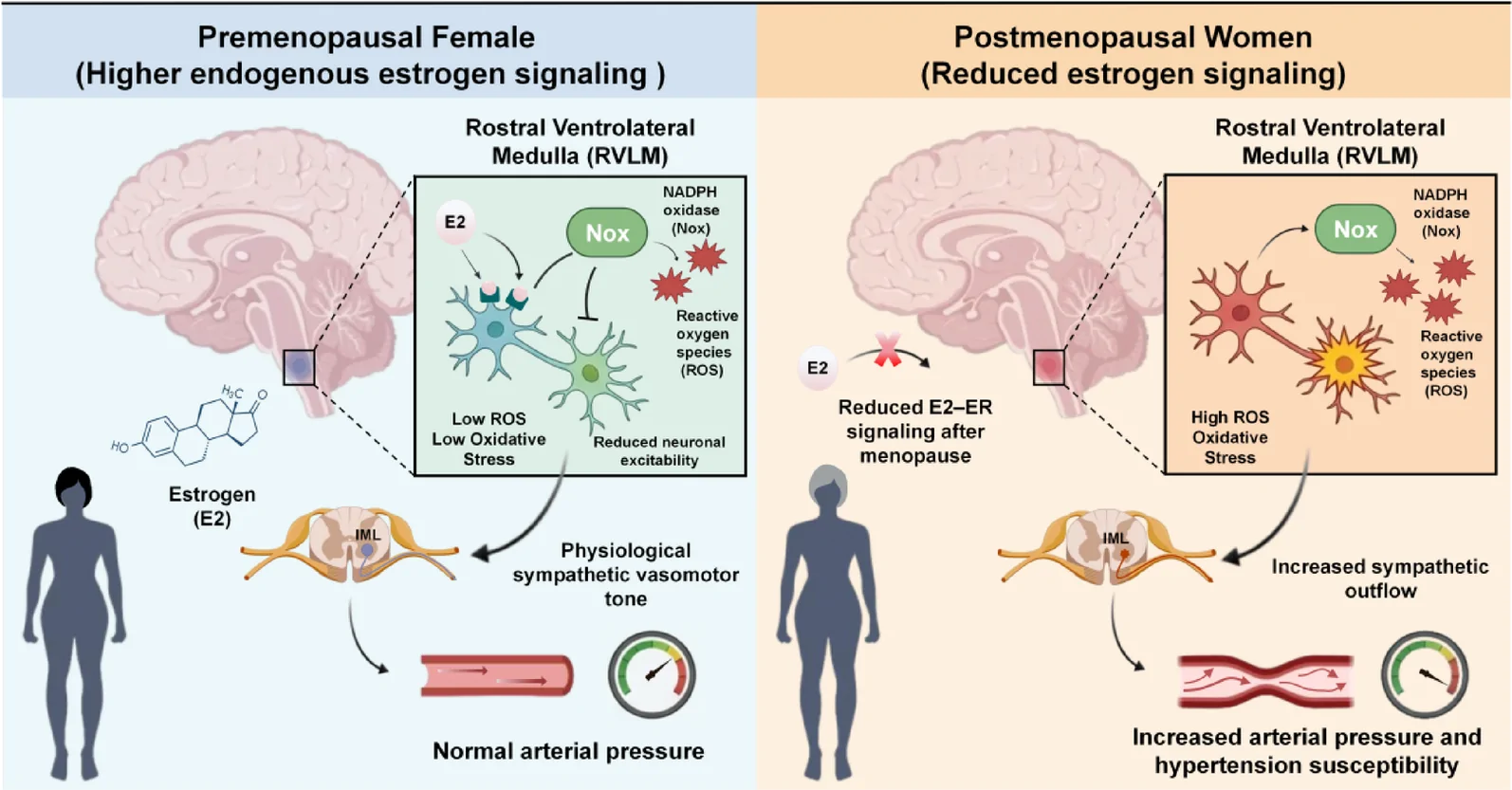
**Proposed changes in estrogen-sensitive RVLM redox regulation before and after menopause. **Higher endogenous E2 signaling before menopause may restrain Nox-derived ROS production and neuronal excitability in the rostral ventrolateral medulla (RVLM), thereby supporting physiological sympathetic vasomotor tone. After menopause, reduced estrogen-sensitive signaling may favor increased RVLM oxidative stress and sympathetic outflow, contributing to greater susceptibility to elevated arterial pressure. This conceptual diagram integrates clinical evidence of menopause-related changes in blood pressure and autonomic regulation with preclinical evidence concerning estrogen signaling, RVLM oxidative stress, and bulbospinal sympathetic pathways [9-15, 24-27, 99, 101, 105, 111]. The complete mechanism has not been directly demonstrated in women. Created by the authors based on the cited studies. The graphical tool for authors to generate the figure is BioGDP [33]

Figure 3 summarizes a proposed framework through which menopause-associated reductions in estrogen-sensitive signaling may influence RVLM redox regulation, sympathetic outflow, and susceptibility to elevated arterial pressure.

### Baroreflex and autonomic regulation across the menopausal transition

Menopause is accompanied by changes in sympathetic activity and baroreflex regulation. Postmenopausal women display stronger sympathetic cardiovascular responses at rest and during physiological stress compared with age-matched premenopausal women [114]. Because baroreflex control includes cardiovagal regulation of heart rate, sympathetic regulation of peripheral resistance, and vascular responses to sympathetic discharge, its individual components may change in different directions [115].

In early postmenopausal women without hypertension, baroreflex sensitivity was lower than in BMI-matched premenopausal women and was accompanied by reduced vagal indices, a higher LF/HF ratio, higher blood pressure, and associations with inflammation, oxidative stress, and prehypertension [109]. Microneurographic studies similarly found increased resting muscle sympathetic nerve activity and reduced cardiovagal baroreflex sensitivity. However, spontaneous sympathetic baroreflex sensitivity was enhanced while the ability of sympathetic bursts to increase blood pressure was reduced, possibly reflecting compensation for reduced vascular responsiveness [116].

Preclinical evidence supports the contribution of ovarian hormone status to autonomic control. Ovarian hormone-deprived rats showed higher arterial pressure, greater cardiac sympathovagal balance, increased pressure variability and oxidative stress, and impaired baroreflex sensitivity [117]. In ovariectomized SHR, exercise training, alone or combined with hydrochlorothiazide, improved bradycardic and tachycardic baroreflex responses, whereas hydrochlorothiazide alone did not restore baroreflex sensitivity. Combined treatment also improved sympathetic, inflammatory, and redox indices [118]. However, these studies do not show whether the observed effects occur within the RVLM or involve a specific Nox isoform.

Heart rate variability provides indirect evidence and should not be equated with direct measurement of baroreflex gain. Postmenopausal women showed lower unadjusted baseline heart rate variability and reduced high-frequency power during Ang II challenge, but circulating estradiol was not linearly associated with heart rate variability [119]. Severe vasomotor symptoms have also been associated with greater sympathetic activity and higher blood pressure [120, 121].

Studies comparing women before, during, and after menopause have reported differences in resting sympathetic activity, cardiovagal baroreflex control, sympathetic baroreflex sensitivity, and vascular responses to sympathetic nerve activity [108]. Current clinical measurements cannot determine whether these differences arise from the RVLM, PVN, NTS, peripheral vasculature, or interactions among these sites.

### Insights and limitations from menopausal hormone therapy

MHT provides clinically relevant but mechanistically complex evidence regarding cardiovascular and autonomic regulation after menopause. MHT is not equivalent to endogenous 17β-estradiol signaling, and clinical regimens differ in estrogen formulation (oral or transdermal delivery) , dose, treatment duration, and accompanying progestogen. Oral estrogen undergoes hepatic first-pass metabolism and produces a different circulating estrogen profile from transdermal estradiol, while progestogens may further modify cardiovascular responses [122]. Clinical findings must therefore be interpreted according to the specific treatment and population.

Direct autonomic studies have not produced uniform results. Short-term transdermal estradiol reduced resting muscle sympathetic nerve activity in healthy postmenopausal women without significantly changing blood pressure or heart rate [123]. In a small randomized crossover study, eight weeks of transdermal estradiol reduced sympathetic nerve discharge and modestly lowered ambulatory diastolic pressure, whereas oral conjugated estrogens did not produce a comparable effect [124]. A later randomized trial found that oral estradiol valerate did not reduce muscle sympathetic nerve activity and prevented the reduction associated with aerobic training [125]. These findings suggest that transdermal estradiol may reduce sympathetic activity in selected populations, but they do not establish a consistent autonomic effect across MHT regimens. Preclinical RVLM studies likewise indicate that endocrine context influences responses to estradiol, although these models cannot directly predict clinical MHT outcomes [10, 12, 13].

Large cardiovascular trials further illustrate the importance of treatment context. HERS did not support combined conjugated equine estrogen and medroxyprogesterone acetate for secondary prevention of coronary heart disease, while the Women’s Health Initiative did not support the same regimen for primary cardiovascular prevention in the population studied [126, 127]. KEEPS did not demonstrate slower carotid intima–media thickness progression with either oral conjugated estrogens or transdermal estradiol in recently menopausal women. By contrast, ELITE reported slower progression when oral estradiol was initiated within six years after menopause, but not when treatment began ten or more years after menopause [128, 129]. Differences in study population, baseline cardiovascular risk, estrogen formulation, administration route, progestogen exposure, and clinical endpoint prevent these findings from being attributed to treatment timing alone or summarized as a uniformly beneficial or harmful effect.

Clinical autonomic and vascular measurements cannot localize treatment effects to the RVLM or reliably distinguish central from peripheral mechanisms. Effective hormone exposure within the central nervous system may also vary with administration route, plasma protein binding, local steroid metabolism, receptor distribution, and conversion between estradiol and estrone [122]. Clinical MHT studies therefore provide important translational context but do not directly establish signaling between estrogen receptors and Nox isoforms within the human RVLM.

## Conclusion and future directions

### Conclusions

The RVLM is an important sympathetic premotor region within the central autonomic network, integrating reflex, hypothalamic, neurohumoral, inflammatory, and metabolic inputs into vasomotor sympathetic output. Oxidative stress within this region can increase neuronal excitability, strengthen glutamatergic transmission, weaken nitric oxide-dependent GABAergic inhibition, and promote sympathetic activation. Direct RVLM evidence is strongest for components of the NOX2 complex, including gp91phox, p22phox, and p47phox, whereas the cellular localization and functional roles of Nox1, Nox4, and other isoforms remain less clearly defined. RVLM Nox-related oxidative stress appears to contribute mainly to the amplification and maintenance of hypertension.

Estrogen modifies RVLM redox balance and sympathetic regulation, but its effects depend on the endocrine and experimental context. Estradiol replacement after ovarian hormone loss can reduce RVLM ROS and NOX4 and improve antioxidant and sympathetic regulation [10], whereas prolonged additional estradiol exposure in intact cycling females can increase RVLM superoxide and transcription related to Nox1 and Nox2 [12, 13]. These findings arise from biologically different conditions and may vary with ligand, dose, duration, receptor subtype, age, central exposure, and hypertensive background. They therefore do not support a uniformly protective or uniformly prooxidant model of estrogen action.

Human studies associate menopause with changes in blood pressure, arterial stiffness, sympathetic activity, and baroreflex regulation, while the cardiovascular effects of MHT vary with formulation, route, treatment timing, progestogen exposure, and patient characteristics. These systemic findings define clinically relevant associations but provide no region-specific evidence for signaling between particular estrogen receptors and Nox isoforms within the human RVLM. The available evidence consequently supports a conditional framework linking biological sex, endocrine state, RVLM redox regulation, and sympathetic output. Direct human causal mapping remains a future objective.

### Future directions

Future studies should define the cellular and subcellular distribution of ERα, ERβ, GPER, and individual Nox isoforms within the RVLM. Particular attention should be given to C1 and non-C1 presympathetic neurons, other neuronal populations, astrocytes, microglia, and local vascular cells. Receptor and Nox localization should be linked to measurements of mRNA, protein abundance, intracellular trafficking, oxidase complex assembly, catalytic activity, specific ROS products, neuronal excitability, sympathetic nerve activity, and arterial pressure. Changes in Nox1 or Nox2 transcripts should be interpreted separately from direct measurements of oxidase activity.

Experimental comparisons should distinguish intact cycling females, ovariectomized animals, estradiol replacement after ovariectomy, reproductive aging or ovarian failure, males, and different hypertensive models. Estradiol formulation, dose, route, duration, treatment timing, circulating and brain hormone concentrations, and selective activation of ERα, ERβ, or GPER should be controlled. The possible actions of estrone, estriol, catechol estrogens, and methoxy estrogen metabolites also require direct investigation. Such designs are necessary to determine whether divergent findings reflect endocrine state, exposure pattern, receptor use, age, or disease background.

Potential estrogen-related interventions remain uncertain. Aromatase inhibitors have no established antihypertensive role, and treatment has been associated with a nonsignificant increase in estimated hypertension risk [130]. Raloxifene and other selective estrogen receptor modulators have generally shown neutral blood pressure effects [131], but the antihypertensive evidence for 2-methoxyestradiol remains preclinical and RVLM involvement has not been tested [132]. Resveratrol can reduce RVLM superoxide in a chronic estradiol exposure model [12] and influence oxidative signaling in the PVN and peripheral vessels [133–135]. Its multiple sites of action currently support a broad redox-modulatory effect rather than a validated RVLM-targeted therapy.

Approaches targeting Nox also remain exploratory. In spontaneously hypertensive rats, the dual Nox1 and Nox4 inhibitor GKT137831 reduced ROS production and inflammatory signaling and attenuated cardiac hypertrophy [136]. However, these effects were demonstrated in peripheral cardiac tissue, and whether Nox inhibitors can reach the RVLM and reduce sympathetic activity remains to be established. Regulators of oxidase maturation or assembly, including EROS/CYBC1 and PDIA1, may provide additional mechanistic targets, although current evidence derives mainly from immune or vascular cells rather than the RVLM [137–141]. Clinical translation will require evidence of CNS bioavailability, regional and cellular target engagement, Nox isoform selectivity, long-term safety, sustained effects on sympathetic activity and blood pressure, and separation of central from peripheral actions. Stratification by menopausal stage, time since menopause, sympathetic activity, baroreflex function, arterial stiffness, metabolic status, and hormone treatment may eventually identify relevant patient subgroups. At present, central redox modulation is a hypothesis-driven translational direction that requires these preclinical and clinical validation steps before therapeutic application.

## Data Availability

No datasets were generated or analysed during the current study.

## Abbreviations

2K1C: Two-kidney, one-clip
ACE: Angiotensin-converting enzyme
AOF: Accelerated ovarian failure
AMPK: AMP-activated protein kinase
AMPA: α-amino-3-hydroxy-5-methyl-4-isoxazolepropionic acid
Ang II: Angiotensin II
AP: Area postrema
ARB: Angiotensin II receptor blocker
AT1R: Angiotensin II type 1 receptor
ATP: Adenosine triphosphate
C/EBPβ: CCAAT/enhancer-binding protein beta
CNS: Central nervous system
COX-2: Cyclooxygenase-2
CRF1: Corticotropin-releasing factor receptor 1
CVLM: Caudal ventrolateral medulla
CVOs: Circumventricular organs
DPI: Diphenyleneiodonium
E2: 17β-estradiol
EGFP: Enhanced green fluorescent protein
ELITE: Early versus late intervention trial with estradiol
eNOS: Endothelial nitric oxide synthase
ERα: Estrogen receptor alpha
ERβ: Estrogen receptor beta
ERK1/2: Extracellular signal-regulated kinases 1 and 2
EROS/CYBC1: Essential for reactive oxygen species/cytochrome b-245 chaperone 1
FOXO1: Forkhead box O1
GABA: γ-aminobutyric acid
GPER: G protein-coupled estrogen receptor
GluA1: Glutamate ionotropic receptor AMPA type subunit 1
GluN1: Glutamate ionotropic receptor NMDA type subunit 1
HERS: Heart and estrogen/progestin replacement study
IL-1β: Interleukin-1 beta
KEEPS: Kronos early estrogen prevention study
Kv4.3: Voltage-gated potassium channel Kv4.3
LF/HF: Low-frequency/high-frequency ratio
MAPK: Mitogen-activated protein kinase
MHT: Menopausal hormone therapy
miR-22-3p: MicroRNA-22-3p
mRNA: Messenger RNA
NADPH: Nicotinamide adenine dinucleotide phosphate
NMDA: N-methyl-D-aspartate
NO: Nitric oxide
NOS: Nitric oxide synthase
Nox: NADPH oxidase
Nox1: NADPH oxidase 1
Nox2/NOX2: NADPH oxidase 2
Nox4/NOX4: NADPH oxidase 4
Nox5: NADPH oxidase 5
NoxO1: NADPH oxidase organizer 1
Nrf2: Nuclear factor erythroid 2-related factor 2
NTS: Nucleus tractus solitarius
OVLT: Organum vasculosum of the lamina terminalis
OX1R: Orexin 1 receptor
P2Y12: P2Y purinergic receptor 12
PDGFB: Platelet-derived growth factor subunit B
PDGFRα: Platelet-derived growth factor receptor alpha
PDIA1: Protein disulfide isomerase A1
PNMT: Phenylethanolamine N-methyltransferase
PVN: Paraventricular nucleus
RAS: Renin–angiotensin system
ROS: Reactive oxygen species
RVLM: Rostral ventrolateral medulla
SFO: Subfornical organ
SHR: Spontaneously hypertensive rats
SHRSP: Stroke-prone spontaneously hypertensive rats
SIRT1: Sirtuin 1
Sirt3: Sirtuin 3
SOD1: Superoxide dismutase 1
TH: Tyrosine hydroxylase
TNF-α: Tumor necrosis factor alpha
TNFR1: Tumor necrosis factor receptor 1
TTX: Tetrodotoxin
VCD: 4-vinylcyclohexene diepoxide
VGLUT2: Vesicular glutamate transporter 2
WKY: Wistar–Kyoto

## Declarations

### Artificial Intelligence Technology

Artificial intelligence was utilized solely to enhance the clarity and quality of English expression during the manuscript drafting process, as well as to assist the authors in searching for and understanding the literature. All content was subsequently reviewed and revised by the authors. Statement: During the preparation of this work, the authors utilised ChatGPT to facilitate reviewing and comprehension the literature. Following the use of this tool, the author conducted the necessary review and editing of the content and assumes full responsibility for the content of the published article.
